# Limitations of lymphoblastoid cell lines for functional analysis of SNPs

**DOI:** 10.1186/s13104-017-2864-6

**Published:** 2017-11-02

**Authors:** Hansi Pathak, Helge Frieling, Mathias Rhein, Alexandra Burkert

**Affiliations:** Laboratory for Molecular Neuroscience, Department of Psychiatry, Social Psychiatry and Psychotherapy, Hannover Medical School (MHH), 30625 Hannover, Germany

**Keywords:** SNP, Epigenetics, LCLs and HapMap

## Abstract

**Objective:**

Single nucleotide polymorphisms (SNPs) are widely linked to the susceptibility and penetrance of diseases. SNP rs886205 (A/G) located in the aldehyde dehydrogenase 2 (ALDH2) promoter is associated with esophageal carcinoma in alcohol-dependent patients. Previously, we found an interaction of the SNP with the methylation of promoter regions as well as the protein levels of ALDH2 in alcohol-dependent patients. To study the DNA–protein interactions involved in rs886205 mediated regulation of ALDH2, we chose lymphoblastoid cell lines harboring AA/GA/GG genotype and acquired two for each genotype from National Human Genome Research Institute repository. We measured the promoter methylation of ALDH2 by using bisulfite sequencing and quantified protein expression of ALDH2 by western blot to compare the cell lines with the previous findings in patients.

**Results:**

DNA methylation showed significant differences not only based on genotype but also due to the different background of the cells owing to their origin from different individuals. Although ALDH2 protein expression seemed to be driven by the rs886205 genotype, results were not in consensus with data from the patient cohorts. Our findings show the limitations of the usage of lymphoblastoid cell lines due to the unavoidable background genetic differences that may influence the effect of SNP.

**Electronic supplementary material:**

The online version of this article (10.1186/s13104-017-2864-6) contains supplementary material, which is available to authorized users.

## Introduction

Investigating genetic variations is important to understand the contribution of inter-individual differences in the manifestation of physiological and psychological disorders [1, 2]. The limbic system plays a central role in mechanisms of addiction towards alcohol, nicotine and psychoactive drugs like heroin and cocaine [3]. Although all these substances induce the reward circuitry in most individuals, only a few get addicted. These observations formed the basis for studies to understand genetic mechanisms of addiction biology.

In the past, numerous studies were performed in various populations and ethnicities leading to the discovery of the multiple SNPs in alcohol metabolizing enzymes [4, 5]. Among these previously reported SNPs, we focused on SNP rs886205 because of prevalence in the German population and its association with esophageal carcinoma in moderate to heavy alcohol consumers [6, 7].

Two important fragments on the ALDH2 promoter: a negative regulatory element [8] and a positive regulatory element harboring a nuclear receptor response element (NRRE) have been reported [9]. Our Previous findings from the methylation analysis in alcohol-dependent patients revealed an interaction between methylation levels and SNP rs886205. The patients with AA genotype and not with AG/GG showed a decrease in mean methylation of negative regulatory fragment from day 1 to day 7 of alcohol withdrawal. Patients with AA genotype show higher ALDH2 protein expression in comparison to GG/AG patients [10]. In the positive regulatory fragment AA genotype patients showed lower mean methylation than AG/GG patients on day one of alcohol withdrawal. The kinetics of mean methylation also differed in the patients with A and G genotype. Luciferase reporter assays using ALDH2 promoter construct showed higher transcriptional activity in the insert with AA genotype as compared to GG genotype [11]. Therefore our aim was to establish an in vitro system to study distinct rs886205 mediated recruitment of methylation machinery under basal conditions as well as in the course of alcohol intoxication.

The NHGRI repository at coriell institute is a collection of lymphoblastoid cell lines (LCLs) from multiple ethnicities used for HapMap Project, HapMap3 Project and 1000 Genomes Project and serves as an important source to study genetic variations in a range of human populations.

We acquired six LCLs, two each consisting of AA, GA or GG at SNP rs886205 position. We incubated the cells with water or various physiological and nonphysiological concentrations of ethanol and characterized the cell lines for ALDH2 promoter methylation in the negative regulatory region, the positive regulatory region and the dense CpG island spanning through the core promoter along with ALDH2 protein expression. Our results in this study serve as a proof of principle for considering inter-individual differences as an essential parameter before using the LCLs for studies like SNP-mediated gene expression analysis or epigenetic modifications.

## Main text

### Methods

#### Cell culture

The LCLs (established by Epstein–Barr virus transformation of peripheral blood mononuclear cells) were acquired from Coriell biorepositories [cat. no.:GM07048 (AA1), GM07051 (AA2), GM19685 (GG1), GM20814 (GG2), GM07347 (GA1), GM07357 (GA2)] and cultured in RPMI-1640 (Merck Millipore, cat. no. FG1215) medium supplemented with 15% FBS (Gibco^®^, cat no. 10082319) and 1% penicillin–streptomycin (PAN-Biotech, cat. no. P06-07100) at 37 °C with 5% CO_2_. Cells of all three genotypes were seeded in 12-well plates and cultured in PVC chambers filled with 200 ml of water and ethanol (dilutions ranging from 0.05, 0.1, 0.5 or 1% or 10.85, 21.70, 54.26 or 217.07 mM respectively) on metal plates [12]. Cells were harvested after 48 h for DNA and protein isolation. DNA isolation was done in triplicates, and the experiments were replicated for three independent biological repeats.

#### Bisulfite sequencing

500 ng of DNA was used for bisulfite conversion by EpiTect Bisulfite Kit (Qiagen, cat. no. 59720). Sequences of the primer pairs and melting temperatures (Tm °C) are given in Table 1. The length and specificity of the PCR products were confirmed by agarose gel electrophoresis and subsequently purified by the automated PCR purification Agencourt AMPure XP System (Beckman Coulter, cat. no. A63881). Sequencing was carried out by using the BigDye Terminator v3.1 (Applied Biosystems, cat. no. 4337451). After unincorporated dye terminator removal with CleanSEQ (Beckman Coulter, cat. no. A29154) sequences were detected on a 3500xL Genetic Analyzer (Applied Biosystems cat. no. 4406016).

**Table 1:** Primers

No.	Sequence 5′–3′	Tm (° C)
117	TTTGGTGTTGAAATTAGAGTT	60
118	GAGGTATGGTTGTGTGATTG	60
119	ACTCACTACAAACTCTACCTCC	60
524	GTTAAAGGTATATATTGGGGGT	60
525	TATTGGGGGTTTAATTAAGG	60
529	CTTCCTAAAAACCTACGAAAA	60
846	GTGTTAGGTGGTTTTATTTTTTG	62
847	AAACTACCTCTACCATTCCTC	62

List of the primers used. Primers 117–119 were used to amplify the negative regulatory region; primers 524–529 for core promoter and 846–847 for positive regulatory region amplification

*Tm* melting temperature, *°C* degree Celcius

#### Statistical analysis

The methylation levels of each of the CpGs in the three CpG islands was assessed using the Epigenetic Sequencing Methylation Analysis Software (ESME) [13] which compares each methylation site to the original sequence of the promoter. For methylation analysis, mixed linear models for repeated measurements were performed including the factors: rs886205 genotype, cell lines dependent background differences and effect of ethanol incubation at the indicated concentrations. P values of less than 0.05 were considered to indicate statistical significance. Data were analyzed using IBM SPSS Statistics for Windows, Version 21.0 (Armonk, NY: IBM Corp.).

#### Western blotting

25 µg of protein samples were used for SDS-PAGE (BioRad) and were transferred to nitrocellulose membrane. The membrane was blocked with Tris base solution with 1% Tween (TBST) containing 5% non-fat dry milk (NFDM) for 1 h. The membrane was incubated with rabbit anti-ALDH2 (Protein tech, cat. no. 15310-1-AP) and goat anti-vinculin (Santa Cruz Biotechnology, cat. no. SC7649) diluted to 1:500 in TBST containing 2.5% NFDM. The membrane was washed three times with dilution buffer and then incubated with HRP-conjugated goat anti-rabbit and donkey anti-goat (Santa Cruz Biotechnology, cat. no. SC2054 and SC2020 respectively) for 45 min at RT. The membrane was washed three times with TBST and then one time with TBS. Chemiluminescence was detected using the Supersignal West Pico kit (Thermo Scientific, cat. no. 34080) on a Versa Doc imaging system.

### Results

#### Effect of rs886205 and ethanol on ALDH2 promoter methylation

We analyzed three CpG islands located in three important regulatory elements defined previously (i) the negative regulatory region includes 11 CpGs spanning from − 948 bp to CpG-790 bp upstream of the ATG-start site, (ii) the positive regulatory region consisting of NRRE and 14 CpGs, reaching from − 437 to − 259 bp and (iii) a dense CpG island around the core promoter with 52 CpGs embedded from − 251 to + 138 bp. For simplicity, we refer to them as negative regulatory, positive regulatory and core promoter fragment from here on. We used linear mixed model computing methylation as a dependent variable, CpGs as repeated measure and CpG position, genotype, cell line (the two cell lines of the same genotype) and ethanol as factors to assess the effect of single CpG methylation, genotype, cell line dependent disparities and ethanol-mediated methylation changes.

**Fig. 1:**
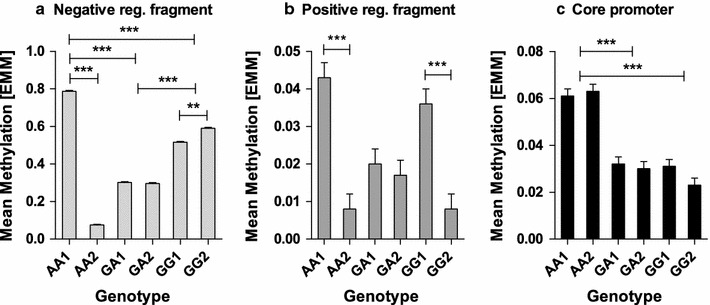
Mean mandhylation of ALDH2 promoter in LCLs of indicated genotype at SNP rs886205. **a** Negative regulatory fragment showed significant differences between AA1 and AA2 cells as well as GG1 and GG2 cells. **b** Cell line mediated differences between AA1 and AA2 as well as GG1 and GG2 were observed in the positive regulatory fragment. **c** Core promoter methylation showed the effect of genotype and AA genotype has significantly higher methylation levels compared to GG or GA genotype. Significant effects are indicated by asterisks (**P ≤ 0.01, and ***P ≤ 0.001). Each bar represents mean ± SEM

*Negative regulatory region* We observed a significant effect of CpG (F _(2, 756)_ = 74.47, P < 0001) and genotype (F _(10, 756)_ = 17.42, P < 0001) and a significant interaction between genotype and CpG position (F _(20, 756)_ = 2.19, P = 002). To assess the differences in the cell lines that may occur owing to their generation from cells isolated from different individuals, we used linear mixed model assigning genotype and cell line as factors and found that methylation differed significantly even though cells were of the same genotype (F _(1, 783)_ = 283.81, P < 0001). We found a significant interaction between genotype and cell lines (F _(2, 783)_ = 368.83, P < 0001). Therefore, we further analyzed the data separately for each genotype and observed a significant difference between the two cell lines of genotype AA and GG (AA1 vs. AA2; (F _(1, 261)_ = 878.68, P < 0001), GG1 vs. GG2; (F _(1, 261)_ = 7.73, P = 006), whereas GA1 and GA2 showed no significant differences in methylation (F _(1, 261)_ = 0.006, P = 0.937) (Fig. 1a). Additionally, we did not observe ethanol-mediated methylation changes on this fragment (F _(4, 3761)_ = 0.359, P = 0.838) (Additional file 1: Figure S1 a).

*Positive regulatory region* A significant effect of CpG position was observed (F _(13, 910)_ = 11.33, P < 0001). Although we do not see an effect of genotype (F _(2, 910)_ = 1.40, P = 0.248), there was an association between genotype and CpG (F _(26, 910)_ = 4.14, P < 0001). We observed cell line mediated methylation changes in between cells of the same genotype (F _(26, 946)_ = 30.98, P < 0001) along with an interaction between cells and genotype (F _(26, 946)_ = 6.355, P = 002). Upon further analysis, we found significant differences between AA1 and AA2 cells (F _(1, 292)_ = 17.69, P < 0001) as well as GG1 and GG2 (F _(1, 334)_ = 17.16, P < 0001). GA1 and GA2 showed similar methylation (F _(1, 320)_ = 0.226, P = 0.635) (Fig. 1b). Estimated mean values illustrated that genotype-mediated differences are nullified as the average methylation levels of AA1 and AA2 as well as of GG1 and GG2 were very similar to GA1 and GA2. We observed a significant effect of ethanol on this fragment (F _(4, 4380)_ = 5.23, P < 0001), but could show no interaction with genotype (F _(8, 4380)_ = 1.11, P = 0.354). Since we observed no concurrence in the genotype-mediated effects as well as no interaction between ethanol and genotype, we did not further analyze the individual concentrations of ethanol in detail (Additional file 1: Figure S1b).

*Core promoter* We observed a significant effect of CpG position (F _(51, 3532)_ = 361.24, P < 0001) and a significant effect of genotype (F _(2, 3532)_ = 147.65, P < 0001) as well as an interaction between the two factors (F _(102, 3532)_ = 12.11, P < 0001). We did not find significant differences between the cells of the same genotype in this fragment (F _(1, 3682)_ = 2.28, P = 0.131) (Fig. 1c). Furthermore we detected no significant effect of ethanol (F _(4, 17732)_ = 1.53, P = 0.190) (Additional file 1: Figure S1c).

#### Effect of rs886205 and ethanol on ALDH2 protein expression

We divided the cell lines into two groups because of technical limitations and therefore compared the effect of genotype and ethanol on cell lines within each group. Group 1 contained the cell lines, AA1, GG1 and GA1 and group 2 contained the cell lines AA2, GG2, and GA2.

**Fig. 2:**
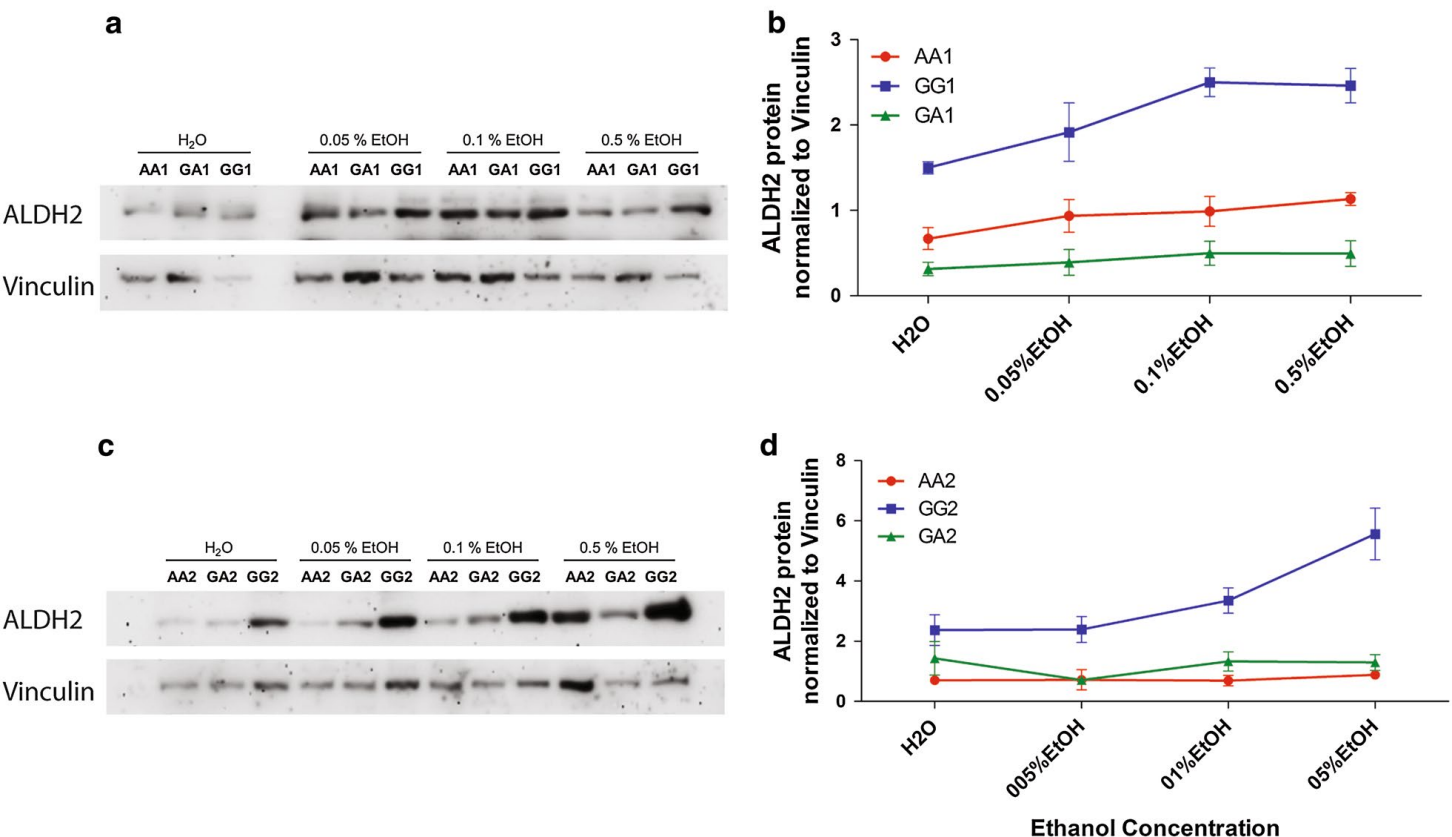
ALDH2 protein expression normalized to Vinculin protein expression in the LCLs of indicated genotype at rs886205 position incubated with H_2_O control or ethanol (0.05% EtOH, 0.1% EtOH or 0.5% EtOH). **a** Representative western blot of group 1 cells **b** and the quantification from three independent experiments. **c** Representative western blot of group 2 cells **d** and the quantification from three independent experiments. GG cells showed significantly higher protein expression compared to AA and GA cells. AA and GA cells did not differ in ALDH2 protein expression. A significant effect of ethanol was observed in both the groups

We observed an overall significant effect of genotype in both the groups; group 1: (F _(2, 24)_ = 100.63, P < 0.0001) and group 2: (F _(2, 33)_ = 46.25, P < 0.0001). Interestingly, GG genotype cells showed higher protein expression in comparison to AA genotype cells, in contrast to our data observed in patients. Further analysis revealed that GG1 differed from AA1 and GA1 significantly in control as well as on ethanol incubation (GG1 vs. AA1: P < 0.01 for H_2_O control and p < 0.001 for 0.05 EtOH, 0.1% EtOH and 5% EtOH; GG1 vs. GA1: p < 0.001 for H_2_O, 0.05% EtOH, 0.1% EtOH and 5% EtOH). GG2 also differed significantly from AA2 and GA2 (GG2 vs. AA2, P < 0.05 for H_2_O control and 0.05% EtOH and P < 0.001 for 0.1% EtOH and 5% EtOH; GG2 vs. GA2: p < 0.05 for 0.05% EtOH, P < 0.01 for 0.1% EtOH and P < 0.001 for 5% EtOH. We found an overall significant effect of ethanol on protein expression in both the groups; group 1: (F _(3, 24)_ = 6.94, P = 0.0025) and group 2: (F _(3, 33)_ = 5.52, P = 0035) but the interaction between ethanol and genotype was only observed in group 2 (F _(6, 33)_ = 5.52, P = 0045). Further analysis showed that this effect was driven by changes in GG2 cells over different concentrations of ethanol (P < 0.001 for H_2_O vs. 0.5% EtOH and 0.05% EtOH vs. 0.5% EtOH; P < 0.01 for 0.1% EtOH vs. 0.5% EtOH) (Fig. 2).

### Discussion

In this study, we characterized the LCLs carrying AA, GG or GA nucleotides at rs886205 position for ALDH2 promoter methylation and protein expression.

Though the methylation status of CpGs in the core promoter confirmed to SNP-mediated changes, negative and positive regulatory promoter fragments showed significant differences in methylation in the cells of the same genotype. AA2 cells showed significantly low methylation compared to AA1 in the negative regulatory fragment as well as positive regulatory fragment and GG2 showed significantly low methylation compared to GG1 cells in the positive regulatory region. To elucidate, if our observations are gene-specific effect or a general defect in methylation machinery, we analyzed promoter methylation of another gene involved in alcohol metabolism, CYP2E1 and found that all the cell lines showed comparable mean methylation (data not shown). We assume that these differences in methylation in regulatory regions might in part be due to the presence of transcription factor binding sites rendering them highly sensitive to the overall differences in the protein expression of important regulatory proteins. In the positive regulatory region, we presumed that these factors are the members of nuclear receptor family on account of the validated NRRE. The presence of putative NF-kappa B consensus sequence has been predicted in the negative regulatory region [8]. As expected the core transcription machinery does not differ in various cell lines and therefore we see a clear genotype dependent difference in the mean methylation.

Although the protein expression in both the cell lines of the same genotype (AA1/AA2; GG1/GG2 and GA1/GA2) shows a similar pattern, it differs from what we have observed in the patients. The difference in the penetrance and random genotype independent methylation differences in positive and negative regulatory fragment suggests that cells from Coriell Institute Repository can differ based on the fragment analyzed and the gene of interest.

Although it is convenient to acquire the cell types of desired SNPs from the repository, these findings argue for the significance of genetic backgrounds of LCLs. Based on our study, we suggest experiments be performed simultaneously in two or more cell lines to ensure scientific rigor in results obtained and ensure that observations made are driven by the genotype of interest.

### Limitations

The alcohol incubation of the cells in chambers is an artificial system and might not replicate the physiological conditions.

## Additional file

**Additional file 1: Figure S1.** Effect of ethanol incubation on mean methylation of ALDH2 promoter. a) No significant effect of ethanol incubation on methylation of negative regulatory fragment. b) We observed significant changes in methylation of positive regulatory fragment upon incubation of cells with ethanol but no interaction with genotype at any of the concentrations. c) No significant of ethanol incubation on methylation of core promoter.

## Abbreviations

ALDH2: alcohol dehydrogenase 2
DNMT: DNA methyltransferase
Erα: estrogen receptor alpha
LCL: lymphoblastoid cell lines
NHGRI: National Human Genome Research Institute
NRRE: nuclear receptor response element
RARα: retinoic acid receptor alpha
RXRα: retinoid x receptor alpha
SNP: single nucleotide polymorphysim
