# Supplementary material for: Limitations of lymphoblastoid cell lines for functional analysis of SNPs
Source: BMC Res Notes. 2017 Nov 2;10:548. doi: 10.1186/s13104-017-2864-6 (PMC5667475; doi:10.1186/s13104-017-2864-6)
Supplement: Supplementary file 1 — Additional file 1: Figure S1. Effect of ethanol incubation on mean methylation of ALDH2 promoter. a) No significant effect of ethanol incubation on methylation of negative regulatory fragment. b) We observed significant changes in methylation of positive regulatory fragment upon incubation of cells with ethanol but no interaction with genotype at any of the concentrations. c) No significant of ethanol incubation on methylation of core promoter. [file 13104_2017_2864_MOESM1_ESM.pdf]

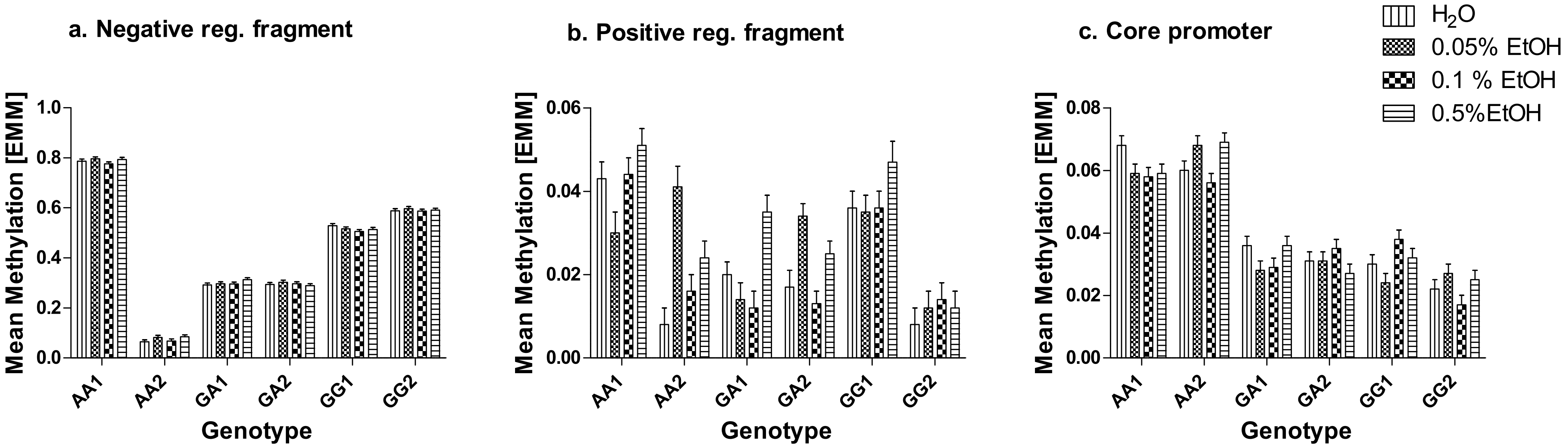

Supplementary Fig 1. Effect of ethanol incubation on mean methylation of ALDH2 promoter.a) No significant effect of ethanol incubation on methylation of negative regulatory fragment. b) We observed significant changes in methylation of positive regulatory fragment upon incubation of cells with ethanol but no interaction with genotype at any of the concentrations. c) No significant of ethanol incubation on methylation of core promoter
